# Molecular Characterization of Fermenting Yeast Species from Fermented* Teff* Dough during Preparation of* Injera* Using ITS DNA Sequence

**DOI:** 10.1155/2019/1291863

**Published:** 2019-07-01

**Authors:** Belay Tilahun Tadesse, Andualem Bahiru Abera, Anteneh Tesfaye Tefera, Diriba Muleta, Zewdu Terefework Alemu, Gary Wessel

**Affiliations:** ^1^Wolkite University, Collage of Natural and Computational Science, Biotechnology Department, Ethiopia; ^2^Addis Ababa Institute of Technology, Addis Ababa University, Ethiopia; ^3^Institute of Biotechnology, Addis Ababa University, Ethiopia; ^4^MRC-ET Molecular Diagnostics, Addis Ababa, Ethiopia; ^5^Department of Molecular Biology, Cellular Biology and Biochemistry, Brown University, Providence, RI 02912, USA

## Abstract

Identification of the yeast responsible for* Injera* fermentation is important in order to be more consistent and for scale-up of* Injera* production. In this study, yeast were isolated and identified from fermenting* teff *dough sample collected from household, hotels, and microenterprises, Addis Ababa. Initially, the yeast obtained from fermenting* teff* dough of different sources were selected on the basis of their CO_2_ production potentials. Its DNA sequencing of isolated yeast identified* Pichia fermentans, Pichia occidentalis, Candida humilis, Saccharomyces cerevisiae, *and* Kazachstania bulderi. *The association of identified yeast to their sources indicated the presence of* Pichia fermentans *in fermenting dough samples collected from all sources whereas* Kazachstania bulderi*,* Saccharomyces cerevisiae,* and* Candida humilis *were shown to be present in samples collected from households, hotels, and microenterprises, respectively. The phenotypes and CO_2_ production potentials of this yeast were also documented. This study has confirmed the presence of different yeast species in the fermentation of* teff* dough and hinted the complex nature of* Injera* dough fermentation.

## 1. Introduction

Fermentation, degradation of organic compounds without net oxidation by the microbial, is an important process in production and preservation of food. It is one of the oldest food-processing technologies known, with some records dating back to 6,000 B.C. [1]. Locally with available raw materials from plant or animal sources, people around the world prepare fermented food and drink either naturally or by the addition of microbial starter cultures using traditional knowledge. Micro-organisms, by virtue of their metabolic activities, transform these raw materials both biochemically (i.e., the nutrients) and organoleptically (i.e., the taste/texture/odor, visual appearance) into appealing products that are culturally acceptable to the maker and consumer, which at the same time have improved shelf-life and safety [2, 3]. In addition, the unique group of microbiota increases the levels of protein, vitamin, essential amino acids, fatty acids, digestibility, and pharmacological values. The importance of microbiota in modern-day life is underlined by the wide spectrum of fermented foods marketed in industrialized and developing countries [2]. This also demonstrates that these products play potential role in securing the food demands of people in developing countries [4].

In developing countries food security is a major challenge for many reasons. A traditional way of food production practice is among the major cause because it results in poor yield, inefficient raw material processing, and loss due to poor management, spoilage, and contamination. This mainly holds true regarding food production practices of traditionally fermented foods. In general, in developing countries, most of the traditional fermented foods are made under primitive conditions of spontaneous (uncontrolled) fermentations of nonsterile state which results in low yield and poor quality food products [5]. The conditions, besides having a positive contribution to be important sources of the natural fermenting microorganisms, unfortunately, are exposing the fermented food products to contaminating and spoiling microorganisms that can survive the fermentation conditions [3] and the major sources of diarrheal disease [6] and food spoilage [7, 8], exacerbating poor socioeconomic conditions and taxing the nutritional requirements of the society.

Yeast are the most important microorganism in fermented foods and beverages such as bread,* Injera*, alcoholic beverages, cheeses, and production of biologically important products like insulin, enzyme, vitamins, and antibiotics [9, 10]. Yeast are unicellular, eukaryotic, and polyphyletic organisms classified in the kingdom fungi. They are ubiquitous and commonly found on fruits, bulla, kocho, vegetables, and other plant materials [11]. However, yeast are highly involved in food fermentations, but information on the dominant yeast species in African fermented foods still needs extra engagement especially in Ethiopian fermented teff dough [12, 13]. Thus, the identity of the proper yeast communities for each fermented product is essential.

In Ethiopia most of the research conducted on traditional fermented food primarily focuses on the determination of the microorganisms which are involved in the fermentation, spoilage, and contamination of the food. Unfortunately, limited research has been done on* Injera*, a fermented teff product of major importance in the nutrition and tradition of Ethiopians. Current research reports are largely limited to only investigating microbial ecology of* Injera* employing only phenotypic characterization [12, 14–17]. A very limited literature is available on identification of yeast in teff dough fermentation using molecular approach [18, 19]. This research work aims to identify yeast using molecular approaches to gain a rapid and accurate identification of yeast using intertranscribed spacer (ITS) DNA of yeast [20]. We anticipate this better understanding of yeast microbial communities found in fermented* teff *dough will permit crafting of unique characteristics of* Injera* to enhance its consumption and efficiency in production world-wide.

## 2. Materials and Methods

### 2.1. Sample Collection and Description of the Study Sites

Teff dough samples (two hundred gram each, 97 total) were collected from hotels, households and* Injera* baking microenterprises in Addis Ababa, Ethiopia. Sample processing, laboratory isolation, and identification of yeast were carried out in the Holeta Biotechnology Institute, Microbial Biotechnology Laboratory. Molecular characterization was carried out at the MRC-ET Molecular Diagnostics Laboratory, Addis Ababa, Ethiopia and DNA sequencing was performed at Genewiz (South Plainfield, New Jersey, USA).

### 2.2. Isolation and Selection of Yeast

Samples of dough were serial diluted by taking 10 g of dough from each sample into separate flasks with 90 mL sterile 0.1% peptone water and then homogenized. From this preparation, 1mL was diluted in to 9 ml sterile distilled water; then 0.1mL of appropriate dilution of separate samples was spread onto presolidified Yeast Extract Glucose Chloramphenicol Bromophenol Blue Agar plates (i.e., yeast extract (5.0g/L), glucose (20.0g/L) Chloramphenicol (0.1g/L), Bromophenol blue (0.01g/L)) and incubated at 37°C for 24h. After incubation, representative yeast colonies were selected and subcultured using the same media three times to purify the isolates. Based on their cultural characteristics (colony size, colony color, and colony texture) 155 yeast isolates were selected from each starting culture. Gas (CO_2_) production from glucose was used as a preliminary selection test criterion to further screen the isolates [21].

### 2.3. Production of Gas (CO_2_) from Glucose

A total of 155 yeast isolates were inoculated into tubes containing yeast extract (5.0g/L) glucose (20.0g/L) broth and were incubated at 37°C for 24h. Production of gas (CO_2_) from glucose was assayed using 1.5% peptone water to which 1% glucose was 20.0g/L and 1mL of fresh Andrade indicator solution per 100 mL distilled water was added. The medium was distributed in test tubes containing inverted Durham tubes. The medium was autoclaved at 121°C for 15 minutes and was then inoculated with the required cultures. Sterile paraffin wax of 2 ml was overlaid in each test tube to prevent the entry of oxygen. All tubes were incubated at 37°C and were examined daily for gas production for 7 days [22].

### 2.4. Molecular Characterization of Microbes

#### 2.4.1. Genomic DNA Extraction

The genomic DNA of isolates was extracted using an Invisorb® Spin Genomic DNA Extraction kit according to the instruction of the manufacturer [22, 23].

#### 2.4.2. Amplification of ITS DNA of Yeast Isolates

Regions of each yeast internal transcribed spacers (ITS1 and ITS2) of DNA extract was amplified using nested primers ITS1 and ITS2 [16]. For ITS DNA region, PCR amplification reaction mixtures (50*μ*L) contained 1*μ*L of the extracted DNA, 1*μ*L dNTPs, 1*μ*L of each of primers ITS1 (-TCC GTA GGT GAA CCT GCG G-) and ITS2 (-GCT GCG TTC TTC ATC GAT GC-), 1*μ*L of* Taq* DNA polymerase (1.25 units*/*50 *μ*L) (Fermentas, St. Leon- Rot, Germany), and 5*μ*L PCR buffer. The final reaction volume was adjusted to 50*μ*l by adding reverse osmosis purified water. The PCR cycle conditions include an initial denaturation step at 95°C for 10min, followed by 35 cycles of denaturation at 94°C for 30Sec, primer annealing at 55°C for 1min, and primer extension at 72°C for 1min with a final extension at 72°C for 5min [16]. PCR product then was separated by gel electrophoresis using a 3% Agarose gel and 1*μ*L loading dye with 5*μ*L PCR products and stained with Ethidium bromide for gel documentation.

#### 2.4.3. Cloning and Sequencing of ITS DNA of Yeast

The PCR amplified a genomic region of interest (i.e., ITS DNA of yeast) of each isolate was cloned into pGEMT EZ vector, transformed into XL1-blue cells, and inoculated onto plated LB medium containing 100*μ*g/mL ampicillin for selection according to the manufacturer's instructions (Promega Corporation). Isolates were grown for 16h at 37°C with vigorous shaking in LB/Amp and the resulting plasmids were isolated as described in the QIA quick Gel Extraction Kit. DNA was then sequenced by automated DNA sequencer (ABI model 377; Applied Biosystems) at Genewiz (South Plainfield, NJ USA).

#### 2.4.4. Phylogenetic Analysis

DNA sequences were edited, and consensus sequences were obtained using the Bioedit software package http://www.mbio.ncsu.edu/BioEdit/bioedit.html. Final sequences were then aligned using CLUSTAL (version: 1.2.4) [24] for each of the sequences. The sequences of yeast isolates of this study were then compared to those in GenBank (National Centre for Biotechnology Information; https://blast.ncbi.nlm.nih.gov/Blast.cgi) using the Basic Local Alignment Search Tool [25] for nucleotide sequences (*blastn*). Evolutionary analyses were conducted using the Maximum Likelihood method based on the Tamura-Nei model by MEGA7 [26, 27].

#### 2.4.5. Isolates Designation

Microbial isolates were designated as follows: AAUYT for yeast isolate followed by numbers and capital letters A, B, and C which represent the range of time of fermentation. A represents 48h, B represents 60h, and C represents 72h of fermentation.

#### 2.4.6. Data Analysis

All data were analyzed by using the statistical data analysis software SPSS 20.0 version. And the phylogenetic tree construction was accomplished by MEGA7 software.

## 3. Results

### 3.1. Isolation of Yeast and Gas Production from Glucose

The yeast isolates (n=155) recovered were observed to have different features of colonial morphology. The yeast isolates showed diverse culture characteristics with regard to color (white, gray pigmentation, or blue), shape (circular or irregular), edge (irregular or smooth), and elevation (flat or raised).

### 3.2. Production of Gas (CO_2_) from Glucose

Upon inoculating the yeast isolates in Yeast Extract Glucose Broth (YEGB) and incubating at 25°C for 24h, from a total of 155 yeast isolates obtained from different fermenting teff dough, only 20 were observed with excellent potential of Gas production from glucose (Table 1).

With the abundance of yeast in relation to sample source most isolates were identified from household and then hotel and followed by microenterprise.* Kazachstania bulderi *were found from samples collected from household;* Saccharomyces cerevisiae *and unidentified yeast isolates were found from samples collected from hotel.* Candida humilis *were found as samples collected from households and Microenterprise whereas* Pichia fermentans *were found from all sites.

The yeast isolates were also related to the age of fermentation inoculum used. Some of the yeast were old, some were fresh, and some were both.* Candida humilis *and* Saccharomyces cerevisiae *was found from samples having fresh inoculum whereas* Kazachstania bulderi *were found from samples containing old inoculum.* Pichia fermentans *and other unidentified yeast isolates were found from samples containing fresh and/or old inoculum.

### 3.3. Molecular Characterization of the Isolates

**Figure 1:**
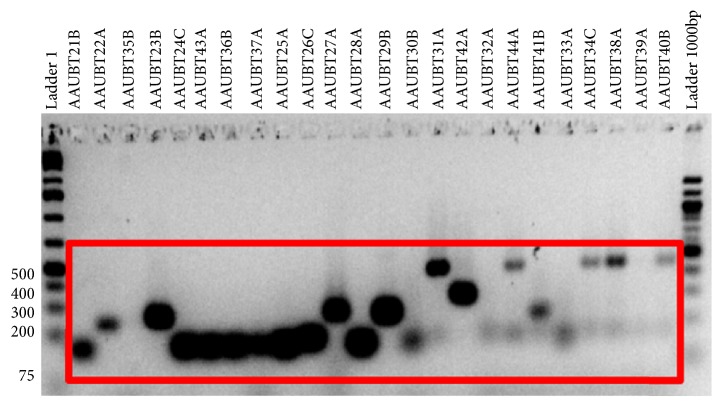
PCR amplification of yeast ITS DNA bands amplified by ITS1 and ITS2 yeast.

The PCR amplification product of 20 yeast isolates of the ITS DNA region has indicated that the yeast isolates recovered in this study have a fragment within 100 to 500bp (Figure 1) which is important for minimizing the number of isolates manageable for sequencing.

#### 3.3.1. Yeast ITS DNA Sequence Analyses

After all the raw sequences were edited, the BLAST result showed that only 13 samples had significant similarity (98-100%) to GenBank entries. Comparisons of ITS DNA sequences of the yeast isolates with yeast found in GenBank permitted the identification of the yeast strains. The ITS DNA sequences of the yeast isolates were used for the construction of the phylogenetic tree of yeast isolated from* teff* dough (Table 2). Sequence similarity was highest (100%) between AAUYT21B and strain of* Pichia fermentans CBS: 2060. *Our result indicated that AAUYT25A and AAUYT27A have 100% similarity with* Pichia fermentans strain Pferm 62_08_02_05.* AAUYT32A with* Pichia fermentans CBS: 4807;* AAUYT23C, AAUYT24C, and AAUYT28A with* Candida humilis CBS: 8195; *AAUYT30B with* Saccharomyces cerevisiae strain YAP1*; and AAUYT26C and AAUYT34C with* Pichia occidentalis strain PMM08-2452L* were found to have 99 % similarity. On the other hand, AAUYT31A was shown to have 98% similarity with* Kazachstania bulderi CBS: 8638*.

Accordingly, in this study the yeast isolates were identified as belonging to four genera* Candida, Kazachstania, Pichia*, and* Saccharomyces.* The genus* Candida *consisted of one species, namely,* Candida humilis CBS: 8195* (isolates AAUYT23B, AAUYT24C, and AAUYT28). The genus* Kazachstania* contained one species called* Kazachstania bulderi *(AAUYT31A), genus* Pichia *contained two species, namely,* Pichia occidentalis *(AAUYT26C and AAUYT34C)*, Pichia fermentans *(AAUYT21B, AAUYT25A, AAUYT27A, and AAUYT32A), and genus* Saccharomyces *contained one species,* Saccharomyces cerevisiae* (AAUYT30B).

**Figure 2:**
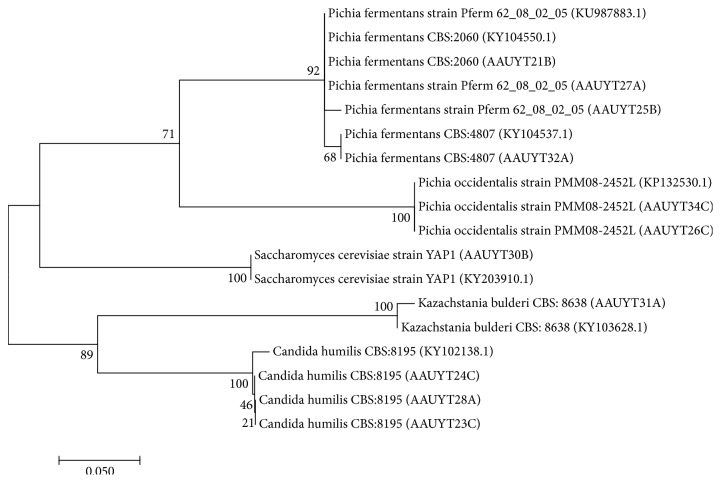
Phylogenetic tree of 13 yeast isolates from fermented* teff* dough using ITS DNA sequence. i.e., 13 yeast isolates were (AAUBT21B, AAUBT25B, AAUBT27A, AAUBT28A, AAUBT24C, AAUBT23C, AAUBT31A, AAUBT26C, AAUBT34C, AAUBT32A and AAUBT30B) and 9 yeast accessions were from GenBank (KY102138.1, KY104888.1, KY104550.1, KU987883.1, KY103628.1, and KY102138.1 AND KY102138.1.).

A phylogenetic tree was constructed from amplified and sequenced ITS DNA region of the 13 yeast isolates of those recovered from fermented* teff* dough in this study, which identified seven species. This phylogenetic grouping indicated that strains having similar sequences were clustered into four genera and nine strains (Figure 2).

The tree was generated through MEGA package version 6 and was used to generate phylogenetic tree based on the Tamura-Nei model for distance estimation and bootstrap analyses with 1,000 replications with a cutoff point of 50 shown next to the branches. The topology of the tree is with similarity cutoff point of 50% and scale length of 0.050 and the highest log likelihood (-430.59). The evolutionary history was inferred by using the Maximum Likelihood method.

## 4. Discussion

This study made a definitive identity of 155 yeast isolates and 20 of these were characterized for their potential in gas production [22]. Yeast isolates with good gas production were believed to be a strong fermenter, preferable for production of* Injera,* and these were selected for molecular identification.

Molecular identification of yeast from fermented* teff* dough using sequence data identified was* Pichia fermentans, Candida humilis, Kazachstania Saccharomyces cerevisiae, *and* Pichia occidentalis*. Mogessie [17] has indicated that yeast found in dough mainly contained* Candida milleri*,* Rhodotorula mucilaginosa*,* Kluyveromyces marxianus, *and* Pichia naganishii. Candida milleri *was found from every household. This independent work supports the previous results of Gifawesen and Abraham [14] who indicated that the dominant yeast of fermenting teff dough were species of Torulopsis*, Saccharomyces, Candida*, and* Pichia*. Askal and Kebede [10] have indicated that* Candida humilis*,* Saccharomyces cerevisiae*,* Cryptococcus tropicalis, *and* Saccharomyces exiguus *were found from early stage to the end of* teff dough* fermentation. In this research also yeast were identified from the beginning of fermentation to the end.

Yeast was identified from all sample sources, i.e., household, hotel, and microenterprises.* Kazachstania bulderi *were found from household whereas* Saccharomyces cerevisiae *and unidentified yeast was found in samples collected from hotels. This variation may be due to personal hygiene and the source of the flour used for dough preparation. Unidentified yeast isolates were found from samples collected from both hotels and households.* Candida humilis *have identified both samples from households and microenterprise whereas* Pichia fermentans *were found from all sites. Yeast was also identified from samples with fresh and old inoculum and the selected isolates were from each category, i.e., samples containing fresh and/ or old inoculum. Berhanu [15] also concluded that yeast become the predominant group of organisms in* ersho* [12].* Saccharomyces *spp. become abundant and is responsible for the rising of the dough. The yeast most prevalent in the* ersho* belonged to the genera* Candida *and* Pichia* [12, 14].* Saccharomyces* spp. and* Torulopsis *spp. are the dominating group of microorganisms after 50h of fermentation [28]. Those identified yeast may involve in* teff* dough fermentation during* Injera* preparation. Knowing the yeast found in* teff* dough at a species level is important for many aspects of the starter culture in formulation for diverse and selected taste characteristics of* teff Injera*.

## 5. Conclusion

The study indicated that there is the involvement of different yeast in teff dough fermentation (i.e.*, Pichia fermentans, Candida humilis, Kazachstania Saccharomyces cerevisiae, *and* Pichia occidentalis*). The identification of these isolates could possibly contribute to the information needed for understanding and verification of potential yeast involved in the course of* teff* dough fermentation.

## Figures and Tables

**Table 1:** Morphological characteristics and gas production of the isolated yeast.

Isolates	Species	Colony Morphology				
		Pigmentation	Shape	Elevation	Size	Gas prod ‘on
AAUYT21B	*Pichia fermentans *	White	Circular	Flat	Large	++
AAUYT23B	*Candida humilis *	White	Circular	Flat	Large	+++
AAUYT24C	*Candida humilis *	White	Circular	Flat	Medium	+++
AAUYT25A	*Pichia fermentans *	White	Circular	Flat	Large	+
AAUYT26C	*Pichia fermentans *	Gray	Irregular	Raised	Large	+
AAUYT27A	*Pichia fermentans *	White	Irregular	Raised	Medium	+
AAUYT28A	*Candida humilis *	White	Irregular	Raised	Large	+
AAUYT30B	*Saccharomyces cerevisiae *	Gray	Irregular	Raised	Large	++
AAUYT31A	*Kazachstania bulderi *	Blue	Circular	Flat	Large	+
AAUYT32A	*Pichia fermentans *	Blue	Circular	Flat	Large	++
AAUYT33A	Unidentified	Blue	Circular	Flat	Large	+
AAUYT34C	*Pichia fermentans *	White	Circular	Flat	Large	+
AAUYT35B	Unidentified	White	Circular	Flat	Large	+
AAUYT36B	Unidentified	Gray	Irregular	Raised	Large	+

**Table 2:** Isolates on the basis of similarity to the partial ITS DNA sequence.

Isolates ID	E value	Identity	Species	Accession
AAUYT23B	0.0	99%	*Candida humilis CBS:8195*	KY102138.1
AAUYT24C	0.0	99%	*Candida humilis CBS:8195*	KY102138.1
AAUYT28A	0.0	99%	*Candida humilis CBS:8195*	KY102138.1
AAUYT31A	0.0	98%	*Kazachstania bulderi CBS: 8638*	KY103628.1
AAUYT21B	0.0	100%	*Pichia fermentans CBS:2060*	KY104550.1
AAUYT25A	0.0	100%	*Pichia fermentans strain Pferm 62_08_02_05*	KU987883.1
AAUYT27A	0.0	100%	*Pichia fermentans strain Pferm 62_08_02_05*	KU987883.1
AAUYT32A	0.0	99%	*Pichia fermentans CBS:4807*	KY104537.1
AAUYT26C	0.0	99%	*Pichia occidentalis strain PMM08-2452L*	KP132530.1
AAUYT34C	0.0	99%	*Pichia occidentalis strain PMM08-2452L*	KP132530.1
AAUYT30B	0.0	99%	*Saccharomyces cerevisiae strain YAP1*	KY203910.1

## Data Availability

The data used to support the findings of this study are available from the corresponding author upon request.

## Abbreviations

bp: Base pair
CO_2_: Carbon dioxide
DNA: Deoxyribonucleic acid
ITS: Internal transcribed spacers
PCR: Polymerase chain reaction
RTA: Ready to prepare
RNA: Ribonucleic acid
*μ*l: Microliter.
